# Detection, Characterization, and Biological Effect of Quorum-Sensing Signaling Molecules in Peanut-Nodulating Bradyrhizobia

**DOI:** 10.3390/s120302851

**Published:** 2012-03-01

**Authors:** Fiorela Nievas, Pablo Bogino, Fernando Sorroche, Walter Giordano

**Affiliations:** Departamento de Biología Molecular, Facultad de Ciencias Exactas, Físico-Químicas y Naturales, Universidad Nacional de Río Cuarto, Río Cuarto, Córdoba, X5804BYA, Argentina; E-Mails: fnievas@exa.unrc.edu.ar (F.N.); pbogino@exa.unrc.edu.ar (P.B.); fsorroche@exa.unrc.edu.ar (F.S.)

**Keywords:** *Bradyrhizobium*, AHLs characterization, biofilm, motility, cell aggregation

## Abstract

Bacteria of the genus *Bradyrhizobium* are able to establish a symbiotic relationship with peanut (*Arachis hypogaea*) root cells and to fix atmospheric nitrogen by converting it to nitrogenous compounds. Quorum sensing (QS) is a cell-cell communication mechanism employed by a variety of bacterial species to coordinate behavior at a community level through regulation of gene expression. The QS process depends on bacterial production of various signaling molecules, among which the N-acylhomoserine lactones (AHLs) are most commonly used by Gram-negative bacteria. Some previous reports have shown the production of QS signaling molecules by various rhizobia, but little is known regarding mechanisms of communication among peanut-nodulating strains. The aims of this study were to identify and characterize QS signals produced by peanut-nodulating bradyrhizobial strains and to evaluate their effects on processes related to cell interaction. Detection of AHLs in 53 rhizobial strains was performed using the biosensor strains *Agrobacterium tumefaciens* NTL4 (pZLR4) and *Chromobacterium violaceum* CV026 for AHLs with long and short acyl chains, respectively. None of the strains screened were found to produce AHLs with short acyl chains, but 14 strains produced AHLs with long acyl chains. These 14 AHL-producing strains were further studied by quantification of β-galactosidase activity levels (AHL-like inducer activity) in NTL4 (pZLR4). Strains displaying moderate to high levels of AHL-like inducer activity were subjected to chemical identification of signaling molecules by high-performance liquid chromatography coupled to mass spectrometry (LC-MS/MS). For each AHL-producing strain, we found at least four different AHLs, corresponding to N-hexanoyl-dl-homoserine lactone (C_6_), N-(3-oxodecanoyl)-l-homoserine lactone (3OC_10_), N-(3-oxododecanoyl)-l-homoserine lactone (3OC_12_), and N-(3-oxotetradecanoyl)-l-homoserine lactone (3OC_14_). Biological roles of 3OC10, 3OC12, and 3OC14 AHLs were evaluated in both AHL-producing and -non-producing peanut-nodulating strains. Bacterial processes related to survival and nodulation, including motility, biofilm formation, and cell aggregation, were affected or modified by the exogenous addition of increasing concentrations of synthetic AHLs. Our results clearly demonstrate the existence of cell communication mechanisms among bradyrhizobial strains symbiotic of peanut. AHLs with long acyl chains appear to be signaling molecules regulating important QS physiological processes in these bacteria.

## 1. Introduction

Bacteria of the genus *Bradyrhizobium* are a diverse group of soil microorganisms that have the ability to establish an association with legume (e.g., soybean, peanut) and non-legume plants (e.g., *Parasponia*) [1]. Peanut (*Arachis hypogaea* L.) is an economically important legume crop cultivated in tropical, subtropical, and temperate areas worldwide. In Argentina, production of peanut is localized in the central region of Córdoba province. Since strains capable of interacting with this legume are genetically highly diverse, the species identity has not been defined for these rhizobia, and the main peanut-nodulating strains are therefore grouped as *Bradyrhizobium* sp. [2,3].

The nitrogen-fixing symbiosis is the result of a complex interaction whereby a plant and a type of bacteria (rhizobia) both obtain nutritional benefit: the bacteria supply the plant with reduced nitrogen from atmospheric sources that are not directly available to the plant, while the bacteria (which would starve in the external soil environment) obtain carbon compounds from the plant within the protected root nodule [4,5]. The shift from free-living soil bacteria to endosymbiont bacteria is a dramatic change that involves physiological, metabolic, and ecological alterations. To undergo this change, rhizobia presumably need to use a chemical communication mechanism to coordinate their activities.

Quorum sensing (QS) is a complex environmental sensing system employed by bacteria to communicate among themselves and thereby regulate their population activities in response to various stimuli. The QS mechanism depends on the synthesis and release of chemical signals into the environment and on the detection of these signals as a function of cell population density. Such group behavior results in altered gene expression that drives the activities of the bacteria in a coordinated manner [6,7].

Bacteria synthesize chemical signals that include a wide variety of small molecules [8]. Of these, the N-acylhomoserine lactones (AHLs) are the most commonly used by Gram-negative bacteria for bacterial communication. The AHL molecule consists of a homoserine lactone ring with an amide-linked acylated side-chain having either a keto or hydroxy substituent at the C_3_ position [9,10]. The biosynthesis and effects of AHLs depend primarily on the activity of the LuxI and LuxR protein families, respectively. After AHLs are produced by LuxI enzymes (AHL synthases), they diffuse across bacterial membranes and accumulate externally until reaching high local concentrations. At a given threshold intracellular concentration, the AHL binds to a LuxR response regulator forming a complex that regulates gene expression [9,11]. AHL-based QS has been shown to be crucial for many plant-associated bacteria, including rhizobia [12–14]. Quorum communication via AHLs in rhizobia affects many metabolic and physiological process, including motility, exopolysaccharide synthesis, biofilm formation, plasmid transfer, root nodulation efficiency, and nitrogen fixing efficiency [15–17].

Most published studies on QS in *Bradyrhizobium* sp. are controversial and restricted to strains symbiotic with soybean. Studies on soybean-nodulating strains have revealed the use of AHL-like signals [18–21], but not in a widespread manner. Loh *et al.* [22] described a mechanism in *Bradyrhizobium japonicum* that depends on cell density and is mediated by a novel signaling molecule named bradyoxetin. Recent studies have demonstrated the production of two new signaling molecules by bacteria of the *Bradyrhizobium* genus: cinnamoyl-homoserine lactone (an aryl-HSL) in photosynthetic stem-nodulating bradyrhizobia [23] and isovaleryl-homoserine lactone (a branched-chain fatty HSL) in the soybean symbiont *Bradyrhizobium japonicum* USDA 110 [24].

A few recent studies have explored QS in *Bradyrhizobium* sp., but none have focused on peanut-nodulating strains. The aims of the present study were to identify and characterize QS signals produced by peanut-nodulating bradyrhizobial strains and to evaluate their effects on bacterial motility and on processes involving cell-cell interaction, e.g., aggregation and biofilm formation.

## 2. Experimental

### 2.1. Bacterial Strains and Culture Conditions

The rhizobial strains used in this study are listed in Table A1. Peanut-nodulating strains were routinely grown on TY medium [25] at 28 °C with rotary shaking (Model SI4-2 Shel Lab, 12 mm orbit, Sheldon Manufacturing Inc., Cornelius, OR, USA) at 150 rpm. *Chromobacterium violaceum* CV026 [26] and *Agrobacterium tumefaciens* NTL4, and their transconjugants *A. tumefaciens* NTL4 (pZLR4) and *A. tumefaciens* NTL4 (pTiC58Δ*acc*R) [27], were grown on LB medium [28] and agrobacterium medium (AB) [29], respectively, at 28 °C with rotary shaking at 150 rpm. When necessary, cycloheximide (50 μg mL^−1^), vancomycin (4 μg mL^−1^), and/or gentamicin (30 μg mL^−1^) were added.

### 2.2. Identification and Quantification of AHLs

#### 2.2.1. Bioassays

For determination of AHL-like molecules with short acyl chains, the biosensor *C. violaceum* CV026 was utilized. This strain is a mini Tn5 double mutant defective in the synthesis of violacein pigment. The production of this pigment *in vitro* is activated by AHLs with short acyl chains. These autoinducers in peanut-nodulating strains were detected by the method of McClean *et al.* [26].

*A. tumefaciens* NTL4 (pZLR4) was utilized to detect AHLs with long acyl chains. This strain carries the plasmid pZLR4, which contains a *traG::lacZ* fusion and *traR*. In the presence of AHLs with long acyl chains the TraR protein is activated, transcription of the *traG::lacZ* fusion is turned on, and LacZ (β-galactosidase) activity can be used as a reporter of *traG* transcription [30]. AHL-like molecules with long acyl chains in peanut-nodulating strains were detected by the method of Farrand *et al.* [31].

#### 2.2.2. Preparation of AHL Extracts

Extraction of AHLs was performed as described by Shaw *et al.* [32]. Peanut-nodulating bacteria were grown to the early stationary phase, and cells were removed from 25 mL growth medium by centrifugation at 12,000 g for 15 min at 4 °C. AHLs were then extracted from culture supernatants with three equal volumes of ethyl acetate, and the extracts were dried and resuspended in 500 μL ethyl actetate.

#### 2.2.3. β-Galactosidase Assay

Aliquots (100 μL) of AHL extracts as above were added to 10 mL cultures of *A. tumefaciens* NTL4 (pZLR4) grown to OD_600_ 0.5. The cultures were incubated for 6–8 h until reaching OD_600_ 1.0. β-galactosidase activity was determined in Miller units as described by Miller [33]. For each strain extract, the values presented are means of four repeated experiments.

#### 2.2.4. Thin-Layer Chromatography

Aliquots (10 μL) of AHL extracts were analyzed by reverse-phase C18-thin layer chromatography (RP-C18 TLC) using methanol/water (60:40 v/v) as the mobile phase. The plates were air-dried, overlaid with AB medium, 0.7% agar containing X-Gal 40 μg mL^−1^, and the biosensor *A. tumefaciens* NTL4 (pZLR4), and incubated overnight at 30 °C [32].

#### 2.2.5. High-Performance Liquid Chromatography and Mass Spectrometry (LC-MS/MS)

AHL extracts were resuspended in 100 μL methanol (100%) for identification and quantification in LC-MS/MS, using a Restek C_18_ (Restek, USA) column (2.1 × 100 mm, 5 μm) with injection volume 10 μL. AHLs were separated at 28 °C using a gradient solvent system with increasing methanol concentration, constant glacial acetic acid concentration 0.2% (v/v) in water, and initial flow rate 0.2 mL min^−1^. The gradient was increased linearly from 40% (v/v) methanol/ 60% (v/v) water–acetic acid to 80% (v/v) methanol/ 20% (v/v) water–acetic acid over 25 min.

Mass spectrometry was performed on a quadrupole tandem mass spectrometer (MS–MS, Quattro Ultima™ *PT*; Micromass, Manchester, UK) equipped with an electrospray ion source (ESI).

AHLs were identified by comparison of retention times and *m/z* transitions with those of the following standards: N-hexanoyl-dl-homoserine lactone (C_6_), N-(3-oxodecanoyl)-l-homoserine lactone (3OC_10_), N-(3-oxododecanoyl)-l-homoserine lactone (3OC_12_), and N-(3-oxotetradecanoyl)-l-homoserine lactone (3OC_14_).

### 2.3. Effect of AHLs on Biological Processes in Peanut-Nodulating Strains

#### 2.3.1. Motility (Swimming) Assay

Bacterial motility was determined in plates containing TY medium and reduced 1/10 TY medium with a final concentration of 0.3% agar. The 3OC_10_, 3OC_12_, and 3OC_14_ AHLs were added at various concentrations. Strains were inoculated by puncture in the center of the plate and incubated for 8 days at 28 °C. Halo diameters as indicators of motility were measured in cm.

#### 2.3.2. Biofilm Formation Assay

Biofilm formation capacity was determined macroscopically by the method of O'Toole and Kolter [34], with some modifications. Glass tubes were inoculated with 800 μL bacterial culture (OD_600_ 0.5) and incubated with agitation for 72 h at 30 °C. Planktonic cells were removed, and each tube was washed three times with saline solution, emptied, stained with crystal violet 0.1% for 15 min, and rinsed three times with distilled water to remove excess crystal violet. Biofilms formed were quantified by adding 1 mL 95% ethanol to the stained tube. The absorbance of solubilized crystal violet was determined by spectrophotometry at 570 nm.

#### 2.3.3. Autoggregation Assay

Each peanut-nodulating strain was grown for 5 days at 28 °C in 25 mL TY medium supplemented with the appropriate AHLs. The bacterial suspension (5 mL) was transferred to a glass tube (10 × 70 mm) and left to settle for 24 h at 4 °C. An aliquot (0.2 mL) of the upper portion of the suspension was carefully transferred to a microtiter plate, and OD_600_ was measured (OD_final_). A control tube was vortexed for 30 s and OD_600_ was determined (OD_initial_). The autoaggregation percentage was calculated as 100[1 – (OD_final_/OD_initial_)] [35].

### 2.4. Statistical Analysis

Experiments on the effects of AHLs on biological processes were conducted using a completely randomized design using at least fifteen (15) replicates for each treatment. The values presented are means of four repeated experiments. The data were subjected to one-way ANOVA followed by comparison of multiple treatment levels with the control using *post hoc* Fisher’s Least Significant Difference (LSD) test. To evaluate the overall effect of AHLs on biological functions, we performed a multivariate study with principal components analysis (PCA). Statistical analyses were performed using Infostat software version 2.0 (Group Infostat, Universidad Nacional de Córdoba, Argentina).

## 3. Results and Discussion

### 3.1. Detection and Characterization of AHL-Like Molecules in Peanut-Nodulating Strains

**Figure 1.**
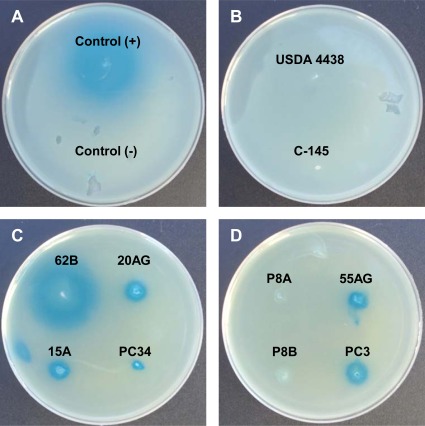
Bioassay for detection of AHL-like molecules with long acyl chains using *A. tumefaciens* NTL4 (pZLR4) as biosensor strain. The plates show bioassay results obtained for control + (*A. tumefaciens* NTL4 pTiC58Δ*accR*), control – (*A. tumefaciens* NTL4) (A), and peanut-nodulating strains (B, C, D). Strains USDA 4438 and C-145 were negative (B); 62B, 20AG, 15A, PC34 (C), 55AG, and PC3 were positive (D); P8A and P8B showed undefined results (D) for AHL production.

AHL-like molecules in peanut-nodulating strains were detected using the biosensor strains *A. tumefaciens* NTL4 (pZLR4) and *C. violaceum* CV026. None of the tested strains showed positive results for the *C. violaceum* CV026 bioassay, indicating that the strains were unable to synthesize AHL-like molecules having short acyl chains. On the other hand, results of the *A. tumefaciens* NTL4 (pZLR4) bioassay revealed that some strains were able to produce AHL-like molecules with long acyl chains. A positive result consisted of the presence of a blue halo around a colony resulting from hydrolysis of X-Gal, as shown in Figure 1. Of the 53 strains analyzed, 14 (26%) were able to synthesize AHL-like molecules with long acyl chains (positive result), two (4%, indicated as +/−) showed an undefined result, and the remaining 37 (70%) showed a negative result for the production of AHL-like molecules by *A. tumefaciens* NTL4 (pZLR4) (Table A1).

The production of AHLs was quantified by measuring β-galactosidase activity using *A. tumefaciens* NTL4 (pZLR4) in rhizobial strains that showed positive or undefined results in the bioassay (Table A1). The response of the strains was quite diverse: five strains showed low levels of AHL production, three strains showed high production, and the remaining strains showed moderate production compared with the control strain (Table A2).

Considering the origin of the strains (Table A1) and their genetic diversity [36,37], our results are in good agreement with those of Pongslip *et al.* [18], who reported the production of long-chain AHLs by ∼22% of a geographically and genetically diverse group of soybean-nodulating bacteria. Similarly, Westenberg [21] reported that three out of 12 tested strains of *B. japonicum* produced AHL-like autoinducers. However, the number of strains evaluated in this study was small, and the biological roles of the autoinducers, if any, were not demonstrated.

**Figure 2.**
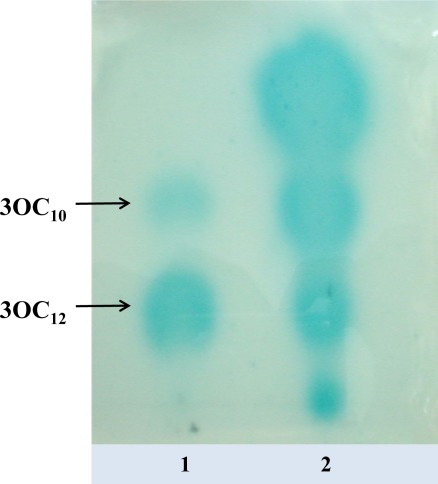
Detection of AHLs with long acyl chain by thin layer chromatography. The TLC plate was overlaid with the biosensor *A. tumefaciens* NTL4 (pZLR4). Lane 1, long chain 3-oxo-standards. Lane 2, 10 μL ethyl acetate extract from supernatant of liquid culture of strain 62B.

Based on the results of the β-galactosidase assay, we selected strains having moderate (62B) and high (P8A) activity for further characterization of the autoinducers by TLC and LC-MS/MS analysis. Using bacterial extracts of strains 62B and P8A, we detected the presence of four different signaling molecules corresponding to standards of C_6_ AHL and 3OC_10_, 3OC_12_, and 3OC_14_ AHLs. Figure 2 shows the TLC patterns of AHLs produced by strain 62B detected by the biosensor *A. tumefaciens* NTL4 (pZLR4). Extracts of strains 62B and P8A (not shown) typically produced four spots detected by NTL4 (pZLR4).

Our results showed clearly that some peanut-nodulating strains had the ability to produce various signaling molecules identified as AHLs with long acyl chains. Similarly, other authors have reported the production of different signaling molecules by rhizobia that nodulate legumes, e.g., *Rhizobium leguminosarum* bv. *viciae* synthesizes mainly C_6_ AHL and 3OC_8_ AHL [38]; *Sinorhizobium meliloti* produces at least six different AHLs, including C_6_ AHL and 3OC_14_ AHL [39].

The analysis of HPLC-MS/MS data allowed us to calculate the concentrations of the molecules produced. In comparison to strain P8A, strain 62B produced higher amounts of all autoinducers, with concentrations of ∼800 nM for C_6_ AHL and 20–30 nM for 3-oxo AHLs. Autoinducer concentrations in P8A extracts were ∼15 nM for C_6_ AHL and 0.1–0.5 nM for the other molecules (Table A3). These findings suggest that the biosensor employed is more sensitive to the AHLs produced by P8A than to those produced by 62B (Table A2). It is possible that the production of different types and concentrations of AHL-like molecules may reflect the physiological regulation of different activities in peanut-nodulating bacterial strains.

### 3.2. Biological Effect of AHLs in Peanut-Nodulating Strains

The biological roles of 3OC_10_, 3OC_12_, and 3OC_14_ AHLs were evaluated in both AHL-producing (62B, P8A) and AHL-non-producing (USDA 4438, P5) peanut-nodulating strains. In view of the lack of information regarding the physiological role of AHLs in various rhizobial processes, we examined the effect of exogenously added synthetic AHLs at various concentrations on activities related to rhizobial survival and nodulation, including motility, biofilm formation, and cell aggregation. To ensure that addition of the solvent (ethyl acetate) employed to dissolve the AHLs did not affect the parameters quantified, we added the same solvent in the control treatments. No effect of the solvent was observed.

#### 3.2.1. Effect of AHLs on Bacterial (Swimming) Motility

Microorganisms with the ability to move have an ecological advantage in that they are able to find more favorable or less hazardous niches for colonizing and persisting in a given environment. In the case of rhizobia, motility has been shown not to be essential for nodulation, but allows the bacteria to find their specific host legume and establish symbiosis. Bacterial motility is an energetically costly process, and therefore must be finely regulated [40]. Several studies using *S. marcescens*, *V. cholerae, E. coli*, and *Y. pseudotuberculosis* have shown that QS systems are involved in the control of bacterial motility [41–44]. We evaluated for the first time the effect of QS signals on the swimming motility of peanut-nodulating bacterial strains. The optimal experimental condition for evaluating the motility of these strains was found to be limited nutrient availability (reduced 1/10 TY medium), whereby their motility was two-fold higher than in full growth medium (entire TY medium) (data not shown). We therefore added the AHLs to reduced 1/10 TY medium and measured the swimming halo of peanut-nodulating strains.

**Figure 3.**
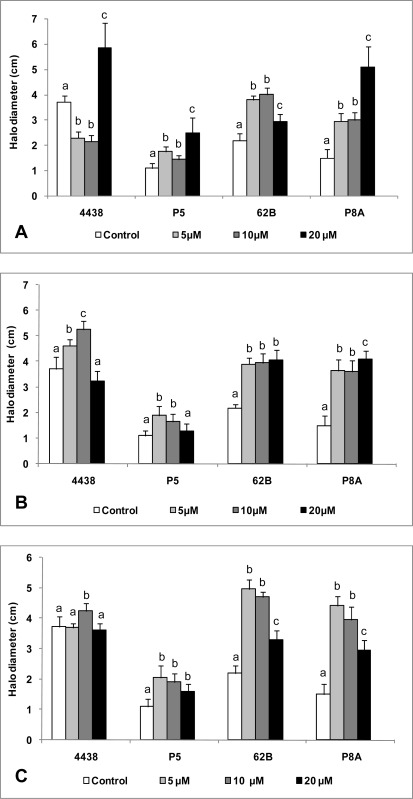
Effect of AHLs on swimming motility of peanut-nodulating strains. Swimming motility (expressed as halo diameter; cm) of peanut-nodulating strains in reduced 1/10 TY medium with 0.3% agar, supplemented with various concentrations of 3OC_10_ AHL (A), 3OC_12_ AHL (B), and 3OC_14_ AHL (C). Values indicated by different letters are significantly different from each other according to Fisher’s LSD test (P < 0.05).

In general, the motility of 62B, P8A, and P5 was increased as a result of exposure to various types and concentrations of AHLs (Figure 3). In the case of 62B and P8A, a low autoinducer concentration (5 μM) resulted in a significant increase in motility, and this effect was maintained or increased in the presence of higher autoinducer concentrations (10 and 20 μM) (Figure 3(A,B,C)). P5 also responded positively to the presence of autoinducers, but to a lesser degree than 62B or P8A. We concluded that, regardless of the type and concentration of autoinducer, these rhizobial strains show increased motility in the presence of exogenous signaling molecules. Interestingly, USDA 4438, a reference strain that did not induce blue haloes in biosensor strains, displayed a more complex swimming behavior when various exogenous AHLs were added. Low concentrations of 3OC_10_ AHL (5 or 10 μM) significantly decreased the motility of USDA 4438, whereas the maximal autoinducer concentration tested (20 μM) significantly increased its swimming ability in comparison to controls without addition of exogenous AHLs (Figure 3(A)). The addition to USDA 4438 of 5 or 10 μM 3OC_12_ AHL caused a slight but significant increase in motility (Figure 3(B)), and the addition of 10 μM 3OC_14_ AHL also caused a slight increase (Figure 3(C)).

Bacterial motility probably occurs as a dispersal strategy in nutrient-limited environments, or to searching for a new host [41–43]. Hoang *et al.* [45] showed that motility of the symbiotic *S. meliloti* is negatively regulated by a QS system, ExpR/SinI. When the bacterial population of *S. meliloti* grows, AHL production increases and motility is repressed. This phenomenon plays an important role in the interaction of *S. meliloti* with its host (alfalfa); *i.e.*, the accumulation of bacteria around the host root and the scope of a quorum could provide an elegant strategy to suppress motility and simultaneously activate the invasion of the root.

There is no previous evidence for a connection between a QS system and motility control in peanut-nodulating *Bradyhizobium* sp. strains. We show here for the first time that the motility process in these bacteria is affected by the presence of QS signaling molecules (AHLs). It is clear that a finely regulated mechanism of cell signaling via AHLs coordinates the process of motility in these bacteria. It remains to be determined whether peanut-nodulating strains employ a motility mechanism for dispersion, colonization of new niches, invasion of the legume host, or other activities.

#### 3.2.2. Effect of AHLs on Biofilm Formation

Bacteria are social organisms that live in terrestrial environments and form highly complex and highly organized communities. Such multicellular structures develop on various surfaces and facilitate survival and optimized resource utilization in hostile environments. This structure and associated lifestyle are termed a “biofilm” [6,46,47]. It has been estimated that over 99% of all bacterial activity in natural ecosystems is associated with bacteria organized in biofilms [48]. Thus, understanding the biology of biofilms is crucial in studies of ecosystems, including those in the soil. QS is a key mechanism that regulates several aspects of biofilm development, including adhesion, motility, maturation, and dispersal [49–52]. The present study is the first to examine the relationship between AHL production and biofilm development in peanut-nodulating bacteria.

**Figure 4.**
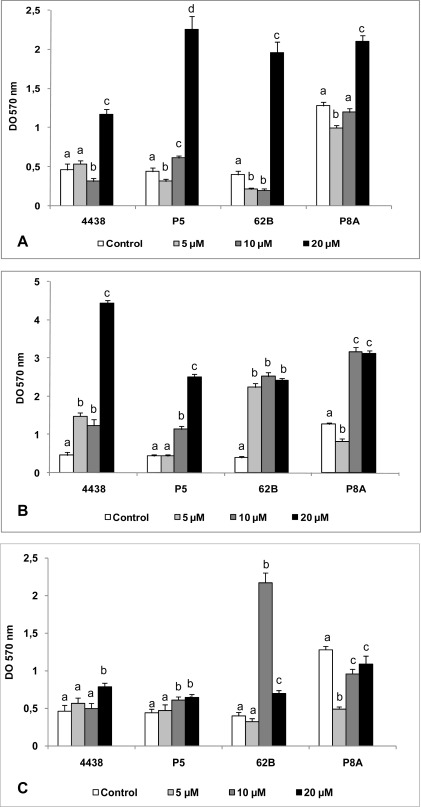
Effect of AHLs on biofilm formation ability of peanut-nodulating strains. The biofilm formation ability of peanut-nodulating strains was determined after 72 h incubation in TY medium supplemented with various concentrations of 3OC_10_ AHL (A), 3OC_12_ AHL (B), and 3OC_14_ AHL (C). Values indicated by different letters are significantly different from each other according to Fisher’s LSD test (P < 0.05).

Regardless of the presence of exogenously-added AHLs, all rhizobial strains tested were able to develop sessile biomass on a glass surface, with OD_570_ values ranging from 0.4 to ∼5.0 (Figure 4). A high concentration (20 μM) of 3OC_10_ AHL caused a significant (3–5 fold) increase in the biofilm formation ability of all strains, relative to strains grown without added AHL (Figure 4(A)). With lower concentrations (5, 10 μM) of added 3OC_10_ AHL, there was either no difference in biofilm formation or a slight inhibitory effect (Figure 4(A)).

In comparison to the results with 3OC_10_ and 3OC_14_, all concentrations of 3OC_12_ AHL tested caused a larger increase in biofilm formation (Figure 4(B)). Similarly to results with 3OC_10_ AHL, a high concentration (20 μM) of 3OC_12_ AHL produced the greatest increase in biofilm formation relative to controls. Such an increase was observed even at low concentrations (5, 10 μM) (Figure 4(B)), indicating that, in contrast to results with other signaling molecules, a significant increase in biofilm formation occurred in the presence of 3OC_12_ AHL regardless of its concentration.

The effect of various concentrations of exogenously added 3OC_14_ AHL was strain-dependent (Figure 4(C)). This molecule only slightly modified biofilm formation by P5 and USDA 4438. Surprisingly, an intermediate concentration (10 μM) of C_14_ AHL greatly increased biofilm formation by 62B strain, whereas all C_14_ AHL concentrations tested reduced biofilm formation by P8A relative to control (Figure 4(C)).

The ability of rhizobia to establish a biofilm can be used as a strategy for survival or for colonization and/or invasion [53,54]. In general, peanut-nodulating strains show increased biofilm formation ability in the presence of added AHLs with long acyl chains, independently of their capacity to synthesize AHLs. However, some strains showed different behaviors depending on the type and concentration of AHL. Since rhizobia are exposed to various and fluctuating environmental conditions, this range of behaviors is presumably employed depending on the prevailing environmental and biological conditions in order to increase rhizosphere fitness and to adapt and survive in a hostile soil ecosystem. The decision to change rhizobial lifestyle from planktonic cell to biofilm cell probably depends on the detection of certain environmental factors that direct the bacteria to activation of cell communication mechanisms that regulate the conduct of the entire population to the new state [6,47,54].

Several recent studies have addressed the regulatory effect of cell signaling mechanisms mediated by AHL on the process of biofilm formation in various Gram-negative bacteria, including *Pseudomonas aeruginosa* [55,56], *Pseudomonas putida* [57], *Serratia liquefaciens* [58], *Aeromonas hydrophila* [59], and *Burkholderia cepacia* [60]. Although there is no direct evidence for a mechanistic relationship between QS and biofilm formation in peanut-nodulating strains, AHLs clearly have an effect on biofilm formation. The ability of some AHL-non-producing strains to form biofilms may be due to the presence of signaling molecules (AHLs or other types) that we were not able to detect. However, there was a clear response to added AHLs by both AHL-producing and AHL-non-producing strains in this study. While there is no previous evidence that a sensing system mediated by AHLs is involved in the mechanism of biofilm formation in peanut-nodulating strains, the present results suggest that these signaling molecules play an important role in the regulation of this process. Such QS regulation may be exerted on surface components such as EPS, LPS, flagella, and pili, which are essential for different steps of biofilm development [54].

It is interesting that putative AHL-non-producing strains are able to respond to the exogenous addition of these signaling molecules. This finding is probably attributable to the ability of strains that are incapable of synthesizing AHLs to recognize them. This could be due to the presence of LuxR-like regulator molecules capable of binding the signaling molecules produced by other bacteria within a given population or community. We hypothesize that this mechanism is used by AHL-non-producing strains and confers an adaptive advantage for regulating certain activities, depending on the bacterial signaling molecules produced by other community members. In nature, biofilms are composed of multiple bacterial species and many other types of organisms, resulting in assemblies that are taxonomically and functionally complex and diverse [61]. Perhaps it is a common occurrence that QS signals produced by certain members regulate the activities of the community as a whole, in ways advantageous for bacterial survival [9].

#### 3.2.3. Effect of AHLs on Cell Aggregation

Cell-cell interactions in bacterial cultures (planktonic cells) are manifested as the autoaggregative phenomenon [62]. Cell aggregation allows the establishment of microcolonies as well as the development of mixed microbial communities in natural habitats. Autoaggregation has been proposed as the initial step in biofilm formation; the cell aggregates formed could then attach to surfaces and develop a sessile population, conferring a competitive advantage and promoting bacterial survival in hostile environments [63,64].

Based on this process of physical cell-cell interaction, there may be mechanisms of cellular communication that mediate the autoaggregative phenomenon. No previous studies have addressed this process or its possible relationship with QS systems in *Bradyrhizobium* sp. We therefore evaluated the effect of AHLs on cell aggregation of peanut-nodulating strains.

**Figure 5.**
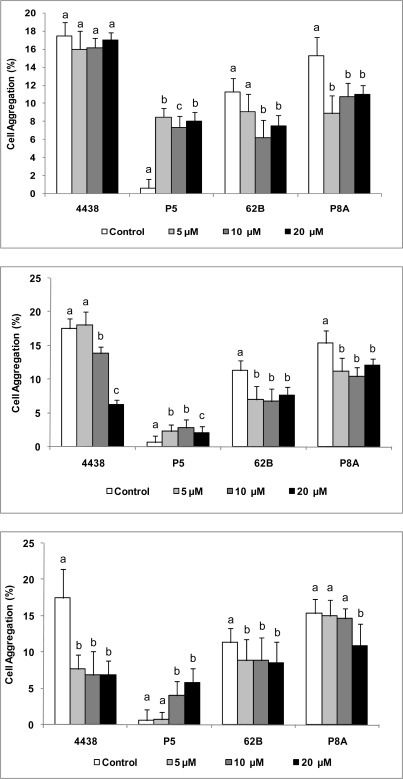
Effect of AHLs on cell aggregation of peanut-nodulating strains. Cell aggregation (%) of peanut-nodulating strains was calculated as described in “Experimental”. For the assay, TY medium was supplemented with various concentrations of 3OC_10_ AHL (A), 3OC_12_ AHL (B), and 3OC_14_ AHL (C). Values indicated by different letters are significantly different from each other according to Fisher’s LSD test (P < 0.05).

Under our experimental conditions, the peanut-nodulating *Bradyrhizobium* strains studied showed a low capacity for cell aggregation, with values ranging from ∼5–20%. In comparison to controls, the addition of exogenous AHLs caused decreased autoaggregative ability in all strains except P5, which showed a slight increase (Figure 5(A,B,C)). All concentration of 3OC_10_ AHL produced the greatest increases in autoaggregation of P5, with values ∼10-fold higher than those of controls (Figure 5(A)). 3OC_12_ (Figure 5(B)) and 3OC_14_ AHLs (Figure 5(C)) showed effects that were similar to but smaller than that of 3OC_10_ AHL. Autoaggregation of USDA 4438 was not affected by addition of 3OC_10_ AHL, but autoaggregation of 62B and P8A decreased in response to increasing concentrations of this autoinducer (Figure 5(A)). A negative dose-dependent effect by 3OC_12_ AHL was observed on autoaggregation of USDA 4438, and a negative dose-independent effect by this autoinducer was observed on autoaggregation of 62B and P8A (Figure 5(B)). Exposure to any concentration of 3OC_14_ AHL significantly reduced autoaggregation of USDA 4438 and 62B, and a high concentration (20 μM) of this autoinducer reduced autoaggregation of P8A (Figure 5(C)).

Previous studies have shown that QS regulatory systems are involved in control of bacterial autoaggregation, e.g., in *Rhodobacter sphaeroides* [65], *Burkholderia thailandensis* [66], *Yersinia pseudotuberculosis* [67], and *Pseudomonas* aureofaciens [68]. Our results indicate that the process of autoaggregation of peanut-nodulating strains is reduced or increased by QS signaling molecules. We can therefore speculate regarding the controlling effect of this process on the behavior of bacterial populations in nature. For some bacterial strains, QS signals may induce the dispersal of aggregates, leading individual bacteria to colonize new microniches in the soil. For other strains, QS mechanisms may positively regulate the autoaggregative process, enhancing the survival of the microorganisms in the soil.

#### 3.2.4. Overall Biological Effect of AHLs on *Bradyrhizobium* Strains

In order to provide a more integrated picture of the impact of AHLs on autoaggregation, biofilm formation, and motility phenotypes of peanut-nodulating strains, we carried out a statistical multivariate PCA. This analysis allowed us to project the data set and variables in a graph in which they could be more easily visualized. The type of AHLs (depending on acyl chain length) associated with the different concentrations tested were the observations (or cases), while the variables were the motility, autoaggregation, and biofilm formation ability of each peanut-nodulating *Bradyrhizobium* strain.

**Figure 6.**
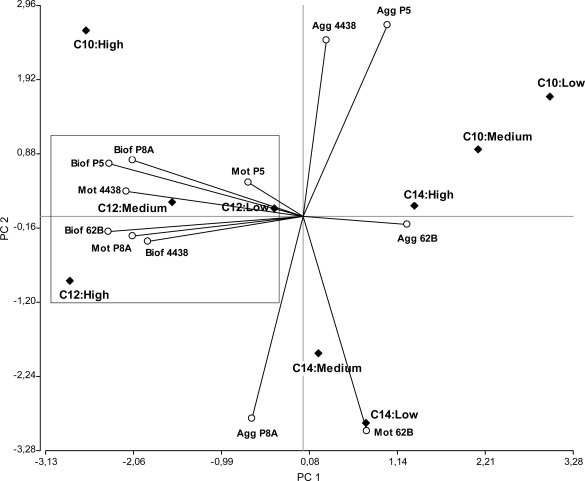
Overall effect of AHLs on various biological processes of peanut-nodulating strains. Graphic obtained from PCA using the software InfoStat version 2.0. Solid diamonds indicate various combinations of AHL type (C10: 3OC_10_; C12: 3OC_12_; C14: 3OC_14_) and concentration (low: 5 μM; medium: 10 μM; high: 20 μM). Open circles indicate the biological variables measured for each strain: motility (Mot), biofilm formation ability (Biof), and cell aggregation (Agg). The angles formed between the straight lines indicate the degree of correlation between variables. PC1: Principal Component 1; PC2: Principal Component 2.

According to the PCA, the analysis of all data in three dimensions (PC1, PC2, and PC3) explained ∼80% of the total variability in the study. The graph generated according to PC1 and PC2 (which explained ∼60% of the variability) (Figure 6) shows that motility and biofilm formation were processes regulated by the influence of 3OC_12_ AHL. Similarly (although not conclusively), it appears that 3OC_10_ and 3OC_14_ AHLs affect the autoaggregative process.

Regarding the correlation between variables, there is a strong positive linkage between the variables of motility and biofilm formation for each strain (acute angle, positive correlation). This is consistent with previous findings that the different mechanisms of bacterial motility play important roles in the colonization of surfaces, which is the first step in the formation of biofilms [69].

Less strongly than for the correlation between motility and biofilm formation, we observed certain linkages between the processes of motility and autoaggregation (acute angles more open). This may reflect the logical necessity of bacterial motility to allow physical contact between the microorganisms prior to cellular aggregation.

In agreement with previous reports [69], motility appears to be a key mechanism for peanut-nodulating bacteria to establish various processes of cell interaction, such as cell aggregation and formation, remodeling, and disassembly of biofilms.

Surprisingly, there were weak or no linkages between biofilm formation and autoaggregation (right or obtuse angles), suggesting that, at least for peanut-nodulating *Bradyrhizobium* strains, these physiological processes may be regulated differently and probably used differently for the bacteria according to their exposure to environmental signals. Thus, the behavior of the bacteria could be driven to aggregation-disaggregation or biofilm formation-biofilm dispersal depending on the type of signal (physical, chemical, *etc.*), the presence-absence of a surface (biotic or abiotic), and other factors. The high biofilm formation capacity (Figure 4) compared to the low autoaggregation percentage (Figure 5) determined for these bacteria suggests that the former mechanism is more important in terms of the environmental benefits that these physiological processes confer to these peanut-symbiotic microorganisms.

## 4. Conclusions

Our results demonstrate for the first time the production of QS signaling molecules by peanut-nodulating bacteria (*Bradyrhizobium* sp. strains). Such molecules were identified as C_6_, 3OC_10_, 3OC_12_, and 3OC_14_ AHLs, and were found only in a small percentage of the bacterial population.

The exposure of both AHL-producing and AHL-non-producing strains to exogenous addition of these molecules had a positive effect on physiological processes related to survival and colonization ability, particularly motility and biofilm formation ability.

In view of the finding that various biological effects observed *in vitro* were affected by different types and concentrations of AHLs, we conclude that the overall behavior of peanut-nodulating soil rhizobial populations is subject to a fine and coordinated regulation of the synthesis of a particular pattern of AHLs, in response to specific environmental factors. We are currently examining the production of AHL-like molecules in the strains that gave negative results in our bioassays, as well as the putative QS mechanism of peanut-nodulating *Bradyrhizobium* sp. The presence of the legume host is presumably a key factor in the regulation of the behavior of rhizobial populations. These hypotheses will be tested in future studies.

## Appendix

**Table A1.** Peanut-nodulating rhizobial strains used, and production of AHL-like molecules with long acyl chains.

**Strain**	**Origin**	**Source**	**AHL**	**Strain**	**Origin**	**Source**	**AHL**
P1	Río Cuarto	[70]	−	35LA	La Aguada	[36]	−
P5	Río Cuarto	[70]	−	51LA	La Aguada	[36]	−
P7	Río Cuarto	[70]	−	61LA	La Aguada	[36]	−
P8A	Río Cuarto	[70]	+/−	65LA	La Aguada	[36]	−
P8B	Río Cuarto	[70]	+/−	36A	La Aguada	This study	+
PR1	Río Cuarto	[70]	−	3A	La Aguada	This study	−
6H	Río Cuarto	[36]	+	4A	La Aguada	[36]	−
PC30	Río Cuarto	[70]	+	8A	La Aguada	[36]	−
PC27	Gral. Cabrera	[37]	+	9A	La Aguada	[36]	−
PC28	Gral. Cabrera	This study	−	15A	La Aguada	This study	+
PC29	Gral. Cabrera	[37]	+	40A	La Aguada	[36]	−
PC31	Gral. Cabrera	[37]	+	Ch1	Chaján	[36]	−
PC34	Gral. Cabrera	[37]	+	Ch4	Chaján	[36]	−
PC3	Gral. Cabrera	[37]	+	Ch8	Chaján	[36]	−
PC4	Gral. Cabrera	[37]	−	Ch9	Chaján	[36]	−
VM45	V. Mackenna	[37]	−	Ch10	Chaján	[36]	−
VM50	V. Mackenna	[37]	−	Ch12	Chaján	[36]	−
V80	V. Mackenna	[37]	−	Ch13	Chaján	[36]	−
62B	V. Mackenna	[37]	+	Ch17	Chaján	[36]	−
20AG	La Aguada	[37]	+	Ch19	Chaján	[36]	−
45AG	La Aguada	[37]	−	Ch21	Chaján	[36]	−
55AG	La Aguada	[37]	+	Ch22	Chaján	This study	−
62AG	La Aguada	[37]	+	Ch25	Chaján	[36]	−
62A	La Aguada	[37]	−	C-145	Reference	NifTAL, USA	−
26LA	La Aguada	[37]	−	USDA 4438	Reference	USDA, ARS, USA	−
27LA	La Aguada	[37]	+	USDA 3180	Reference	USDA, ARS, USA	−
33LA	La Aguada	This study	−				

AHL: Acyl homoserine lactone.

(−): Negative result.

(+): Positive result.

(+/−): Undefined result.

**Table A2.** Induction of β-galactosidase activity in *A. tumefaciens* NTL4 (pZLR4) by AHLs obtained from supernatants of peanut-nodulating rhizobial strains.

**Strain**	**Activity ***
*A. tumefaciens* NTL4 (pTiC58Δ*accR*) ▪	349.6 ± 27.5
*A. tumefaciens* NTL4 ▴	47.0 ± 10.0
*A. tumefaciens* NTL4 (pZLR4) ♦	47.0 ± 5.0

Low AHL-like inducer activity (≤ 100)	

PC29	68.7 ± 16.0
PC27	94.6 ± 6.1
PC30	70.2 ± 12.1
PC34	94.3 ± 8.7
6H	91.9 ± 6.0

Moderate AHL-like inducer activity (≥ 100 < 200)	

15A	123.5 ± 30.3
20AG	129.3 ± 35.8
62AG	109.3 ± 22.6
27LA	101.8 ± 42.6
36A	118.5 ± 17.0
PC3	102.2 ± 12.8
62B	197.1 ± 10.7
55AG	153.9 ± 4.4

High AHL-like inducer activity (≥ 200)	

P8A	369.7 ± 15.5
P8B	411.8 ± 17.1
PC31	678.9 ± 50.4

* β-Gal activity expressed in Miller units;

▪ Control +;

▴ Control −;

♦ Biosensor strain under control conditions (no AHL added).

**Table A3.** AHL concentration (nM) detected by LC-MS/MS for strains 62B and P8A.

	**AHL concentration (nM)**				
**Strain**	**C_6_**	**3OC_10_**	**3OC_12_**	**3OC_14_**
62B	840.1	23.2	28.0	19.6
P8A	15.4	0.13	0.31	0.54
